# A Perspective on Implementing Movement Sonification to Influence Movement (and Eventually Cognitive) Creativity

**DOI:** 10.3389/fpsyg.2020.02233

**Published:** 2020-09-18

**Authors:** Luca Oppici, Emily Frith, James Rudd

**Affiliations:** ^1^Psychology of Learning and Instruction, Department of Psychology, School of Science, Technische Universität Dresden, Dresden, Germany; ^2^Centre for Tactile Internet with Human-in-the-Loop (CeTI), Technische Universität Dresden, Dresden, Germany; ^3^Cognitive Neuroscience of Creativity Laboratory, Department of Psychology, Penn State University, State College, PA, United States; ^4^Research Institute for Sport and Exercise Sciences, Liverpool John Moores University, Liverpool, United Kingdom

**Keywords:** creative cognition, embodied cognition, exercise-cognition, affordance, functional similarity, education, creative

## Abstract

Creativity represents an important feature in a variety of daily-life and domain-specific contexts. Recent evidence indicates that physical movement serves as a key resource for exploring and generating task-relevant creative ideas, supporting the embodied perspective on creative cognition. An intuitive link between movement and creative cognition is movement creativity. The process of exploring the movement solutions an environment offers (i.e., affordances) and exploiting novel, functional, and creative movements may translate to and improve how individuals explore and generate novel ideas. Opening perception to the variety of affordances (“conventional” and novel) an environment offers drives creative movement. Teachers and coaches can promote this process by designing a learning environment that invites performers to consider and utilize novel movement solutions. In this article, we present a rationale for using movement sonification to promote creative movement. Movement sonification consists of mapping a movement parameter into sound, with a sound being triggered or changing according to how movement unfolds. We argue that movement sonification can facilitate the emergence of creative movement *via* enhancing perception of currently performed movements and invite performers to utilize novel affordances, and emphasizing information for regulating subsequent creative actions. We exemplify this concept in a creative dance intervention for children during physical education classes. In conclusion, we contend that learning to explore original dance sequences using movement sonification may provide a meaningful link between creative movement and creative cognition. Children may use their minds *and* bodies as tools for creative thinking and exploration, such as shaping letters with their bodies.

## Introduction

Creativity is a relatively new term with its genesis in the 20th century. In 1968, Wyrick was one of the first to explore an embodied approach to creativity research through emphasizing the importance of movement and its relationship with the environment (Wyrick, 1968). Creative cognition represents an important feature in a variety of daily-life and domain-specific contexts, and the embodied perspective on creative cognition contends that body movement plays a key and active role in the development of creative ideas. An intuitive link between body movement and creative cognition is creative movement. Creative movement is generally defined as a functional and original movement solution to achieve a task goal (Memmert and Perl, 2009; Hristovski et al., 2011), and could be instrumental for creative cognition given its prominence in the art and sport domains (e.g., dance). The process by which creative movement emerges may influence and enhance how creative ideas are generated. Importantly, broadening perception of what the task and environment offers and exploring different solutions to solve a motor task promotes creative movement and can eventually contribute to generating creative ideas. Here, we discuss how the strategy of sonifying a movement – movement sonification – can be used to promote creative movement. While this concept is not novel and sonification has been already used to promote creative movement, primarily in dance improvisation (e.g., Lem et al., 2010; Diniz et al., 2012; Rizzo et al., 2018; Dahlstedt and Dahlstedt, 2019; Erdem et al., 2019), we think that a clear rationale for its implementation in the context of movement creativity is lacking. We present our approach grounded in ecological psychology and discuss how movement sonification can invite performers to explore the variety of movement opportunities (i.e., affordances) the environment offers, thus promoting creativity. Importantly, we also highlight the potential implications that this approach and, more generally, creative movement can have on creative cognition.

## Creative Cognition

Creative cognition is often understood as a collection of mental operations that promote the generation of novel and task‐ or context-relevant ideas (Sternberg and Lubart, 1999; Runco and Jaeger, 2012). Creative thinking is esteemed across many domains, including large-scale scientific achievement, technological innovation, and artistic expression (Cropley, 2006; Moran, 2010). However, small-scale creativity is also an important outlet for self-expression as individuals learn to initiate and pursue novel approaches to everyday problem-solving (Richards, 2010). Practicing everyday creative thinking may be particularly beneficial to the development of a cognitive skillset that lends itself to the fulfillment of creative thinking potential at more impactful levels. This is because, while ability, experience, and capacity indisputably influence the value of creative thoughts, the same cognitive processes are thought to contribute to the production of both seminal and everyday creative ideas (Runco, 2014).

Given the breadth and diversity of creative outcomes, our approach centers on highlighting the role of everyday creative thinking in context (Cropley, 2006; Amabile, 2018). Specifically, several foci of cognitive creativity research encompass strategies for increasing creative thinking in educational contexts (Craft, 2003; Beghetto and Kaufman, 2010; Moran, 2010; Pllana, 2019) by supporting holistic academic success, mental health and well-being, and reinforcing diversity and cross-cultural inclusivity (Lubart and Georgsdottir, 2004; Glaveanu et al., 2019). To this end, it is important to highlight that creative thinking is suggested to be less of an inflexible, enduring personality trait consigned to the minds of geniuses, and is considered more of an externally-modifiable faculty (Amabile, 2018). In other words, creative thinking is proposed to be shaped by both intrinsic factors, including task-relevant skills and motivation, as well as external circumstances, such as affordances and constraints within the task environment (Ward et al., 1999; Amabile, 2018). A context-centered perspective of creative cognition, therefore, permits a broader exploration of the value of everyday creative thinking as a conduit for the construction of meaning across the lifespan.

## The Role of Embodiment in Creative Cognition

Understanding which mental and contextual factors may promote or inhibit creative thinking processes is integral to establishing models that adequately address creative cognition across domains (Ward et al., 1999). Embodied cognition frameworks interleave both mental and physical dynamics of problem-solving, contending that the mind, body, and environment shape the problem/task-goal space, and their interaction guides thought and action that are appropriate to solving the problem (Shapiro and Stolz, 2019). The body may support cognition by offering a means to manipulate and explore the problem-space and reduce cognitive load (Risko and Gilbert, 2016). For example, reading tilted words on a computer screen often requires physical movement (i.e., tilting the head) to accomplish this demanding task, rather than relying solely on mental rotation to match the tilted word stimuli with stored representations of normally-oriented text in semantic memory (Jolicoeur, 1988; Risko and Gilbert, 2016).

The role of movement for creative thinking may be particularly important from a developmental perspective, as a wealth of evidence suggests that early acquisition of motor skills is positively associated with cognitive developments, including memory, language, and problem-solving ability (see Frith et al., 2019). An important mechanism underlying the benefits of movement for cognition is functional similarity between task-relevant movement and cognitive process (Tversky, 2009). Functional similarity between mental and physical operations is thought to scaffold and offload cognition, meaning that the body is a conduit for meaningfully exploring and externalizing task-relevant solutions (both physical and mental). For example, Bara and Bonneton-Botté (2018) demonstrated that movement-based educational programs have the potential to support learning in early childhood compared to sedentary approaches. In this study, kindergarteners were taught to (1) move their arms to draw letters in the air, and (2) walk along letter outlines drawn on the ground. This motor intervention was associated with higher letter recognition and handwriting quality compared to practicing visual recognition of letters and handwriting practice alone. Recent creativity work has also shown that moving *via* gesture (Kirk and Lewis, 2017), and matching (functionally similar) emotional states with physical exertion in dance (Hutton and Sundar, 2010) promoted divergent thinking performance. These findings offer additional credence to the purported role of functional similarity within the mind-body relationship. Taken together, physical movement may serve as a resource for exploring and generating creative ideas and solutions. It is therefore plausible that *creative* movement and *creative* thought processes share functional similarities which reinforce the utility of embodied cognition in this domain.

Practicing and discovering creative movements may further enhance how creative ideas are generated. Building on functional similarity, the process of exploring the movement solutions an environment offers and exploiting novel, functional movements may translate to and improve how individuals explore and generate novel ideas. Indeed, fluid, unstructured and unconstrained (in a way creative) movement has been suggested to serve as a pathway to fluid, distributed thought, which may parallel creative thought processes (Leung et al., 2012; Slepian and Ambady, 2012; Kuo and Yeh, 2016; Zhou et al., 2017). While the link between movement and cognitive creativity has not been thoroughly considered in the literature, in the embodied creativity domain, emphasis should be placed on designing a training environment that offers novel affordances and invites individuals to explore how they might effectively generate creative movement. Considering the prominence of creativity within various physical domains (e.g., dance and sport), we speculate that this approach may have a favorable impact on creative cognition as well.

## Creative Movement

Creative movement is generally defined as a functional and original movement solution to achieve a task goal (Memmert and Perl, 2009; Hristovski et al., 2011; Orth et al., 2017). From an ecological dynamics approach, movement emerges from a continuous, cyclical, and prospective coupling of perception, cognition, and action, situated in the dynamic performer-environment interaction (Gibson, 1979; Davids et al., 1994; Warren, 2006). Humans move to perceive what opportunities for action their environment offers (i.e., affordances), perceive affordances to (self) organize their movement, and, cyclically, movement reveals new (flow of) information that specifies affordances (Michaels and Beek, 1995; Chemero, 2003; Fajen, 2005; Bruineberg and Rietveld, 2014). Across an affordance landscape, some affordances stand out and invite performers to certain actions (Bruineberg and Rietveld, 2014; Rietveld and Kiverstein, 2014; van Dijk and Rietveld, 2016). For example, a variety of actions can be performed in a school gym, but a ball on the ground and a goal create intentionality for most children to perform a kicking action. Creative movement however emerges overtime and from a transformational process, involving search, exploration and discovery of novel, and functionally efficient actions (Hristovski et al., 2009; for an example in dance improvisation, see Kimmel et al., 2018; Rudd et al., 2020). Hypothetically, humans have both opportunities and capacities to perform different creative movements to achieve the same or different goals. In fact, a rich landscape of affordances constantly surrounds a moving organism, offering a vast array of movement options (Bruineberg and Rietveld, 2014; Rietveld and Kiverstein, 2014), and the human body is a multi-stable, degenerate system that can flexibly switch between different movement patterns (Kelso, 2012; Seifert et al., 2013). The more enriched an environment and greater the action capabilities of an individual the higher the possibilities for innovation through interaction creating an abundance of movement options (Bruineberg and Rietveld, 2014; Rietveld and Kiverstein, 2014).

Supporting and teaching creativity to emerge is however a tricky affair as people, typically, are attracted to and utilize affordances to guide their movement that are commonly accepted in their society (Rietveld and Kiverstein, 2014; van Dijk and Rietveld, 2016). In other words, they follow the norm, do what is typically done, and act within their comfort zone. For example, if a teacher turns on the music during a physical education (PE) class and ask children to dance, anecdotally, they will all likely perform a handful of dance movements, which correspond to the current “hits,” e.g., “the floss dance.” Teaching creativity requires designing learning environments that offer a broad range of task-relevant affordances as well as a safe space to encourage an individual to continuously explore functional and novel movement solutions. For example, in teaching the high jump, the introduction of foam-safety mats allowed for safe exploration and practice of landing on the back, which promoted the emergence of a new creative and highly functional movement solution – the “Fosbury Flop.” In this sense, teachers are considered environmental designers that can influence learners’ intention and invite them to explore and discover a range of movement solutions. This safe and non-judgmental (i.e., no correct technique) exploration of an affordance landscape will see individuals experimenting and creating a wide range of movement solutions to the task (Rasmussen et al., 2017; Woods et al., 2020). Keeping with the dance example and pertinent to this paper, the teacher’s instructions should frame a child’s intentionality to be open to new dance movements, explore different movement sequences, and add variability into their movements with the music, and in doing so moving away from the floss dance. Common strategies currently used are instructions (e.g., “avoid imitating your peers” in a class setting) and manipulation of task and environmental constraints (e.g., rules and equipment; Hristovski et al., 2011, 2012; Torrents et al., 2015, 2016). In summary, creative movement emerges when a performer perceives and utilizes novel affordances, and a learning environment (including framing of individual’s intentionality) that encourages perceptual-motor exploration promotes creativity. Here, we provide a theoretical rationale to promote the development of creative movement using movement sonification.

## Movement Sonification

Movement sonification may represent an innovative strategy to enrich a learning environment and promote the development of movement creativity. It consists of mapping a movement parameter into sound, and depending on how the specified movement parameter (s) change (s) a sound is triggered or changes characteristics, e.g., frequency and amplitude (Effenberg, 2005; Hermann et al., 2011; Dyer et al., 2017). For example, a sound tone is triggered when a joint angle exceeds a certain threshold (e.g., Boocock et al., 2019) or a music melody is progressively distorted in reference to the amplitude of a joint angle increase (e.g., Lorenzoni et al., 2019). Given the inherent tight link between movement and sound (Stanton and Spence, 2020), movement sonification has recently gained an increased interest in the motor learning and control field as a suitable strategy to deliver augmented feedback (Sigrist et al., 2013; Dyer et al., 2015). In fact, sonification of a movement parameter has been shown to enhance a multimodal perception of intrinsic feedback (e.g., proprioceptive information) and the dynamics of perception-action coupling (Dyer et al., 2017), typically resulting in improved motor learning and performance (for reviews, see Effenberg et al., 2016; Schaffert et al., 2019). Here, we discuss how movement sonification can also be used to influence movement creativity.

Movement sonification can be used to enhance how a performer perceives the (currently) utilized affordances, directing them to novel affordances, and promote a change in a learner’s intentionality toward an exploration of a new, functional, and creative movement. Once a learner is aware of the currently used affordances and changes their intentionality toward trying out new movements, they start a movement exploration process that will promote the emergence of movement creativity. The exploration process will perturb the performer-environment dynamic (e.g., learner and music) and will shape new affordances for novel creative movement. A learner can spontaneously change their intention (“I hear the sound changing as I change my moves, I should experiment with these movements and sounds”) or teacher’s should educate the learner’s attention toward the environmental shift caused by their movement, thus supporting the learner’s knowledge of the environment (Gibson, 1979). Importantly, movement sonification *per se* does not shape novel affordances but invites learners to explore a broad range of new movements, which in turn will create new affordances. In short, the key component for movement creativity to emerge is a learner’s exploration of movement options, and movement sonification can promote this process. These mechanisms are discussed hereafter, and their application is exemplified in a creative dance intervention for children during PE classes, which represents a suitable learning context for creative movement.

As previously mentioned, a critical component for creative movement to emerge is a learner’s perceptual openness and attunement to the rich landscape of affordances surrounding them (Rietveld and Kiverstein, 2014). A performer should be aware of the currently used affordances and be invited to find new solutions. In this context, movement sonification can enhance one’s awareness of the movement solutions they are currently adopting and support a change in their intentionality toward trying different (functional) movements. Previous research has shown that sonification increased dancers’ awareness of the “movement vocabulary” they were using and movement sequences they were performing and facilitated their exploration of novel movement patterns (Diniz et al., 2012; Françoise et al., 2014; Wood et al., 2017). With this, we are not suggesting that movement sonification should direct a performer’s attentional focus to their movement (which has been shown to be detrimental for motor performance and learning; Wulf, 2013), but instead it should enhance a performer’s perception of how they are currently using the variety of movement possibilities the environment is offering. In short, movement sonification will promote an enhanced performer’s attunement to the dynamics of task-environment they are embedded in. Keeping with the previous dance example, if a PE teacher turns on music and asks their children to create dance moves, they likely will replicate current dance “hits” (i.e., a handful of movements). To encourage children to find new movement, some parameter of the music (such as frequency and tempo) can be mapped onto children’s movement and change according to how they find new movements. An initial assessment of children’s typical dance moves is needed to set a child’s movement signature as reference, and a selected music parameter can change when child deviates from their movement signature. If necessary, to educate a child’s attention toward knowledge of the environment, the teacher could briefly explain how a child’s movement can change music, and invite their students to explore movements to manipulate and play with the speed and tempo of the music through their movements. This will enhance children’s perception of their currently adopted movement (i.e., music does not change if they perform the usual movement) and invite them to try new movement (i.e., music changes).

Movement sonification is mapped within the coupling of perception and action, and represents an informational constraint that can facilitate releasing a movement’s degrees of freedom (hence creativity, see Hristovski et al., 2011; Torrents et al., 2020). From the cyclical coupling of perception and action, action “creates” new information for further action, and sonification can amplify this newly “created” information and encourage learners to perceive and exploit this information. This can be particularly relevant for sequences of movements and movement improvisation (e.g., in dance), whereby each movement is regulated on the (information about) previous movement. In this sense, movement sonification facilitates a learner’s perception of the “novel” affordances. Previous research in dance improvisation has shown that sonification enhanced participants’ variety of novel movements relative to a no-sonification condition (Yamaguchi and Kadone, 2017) and supported the creation of Japanese dance sequences (Dahlstedt and Dahlstedt, 2019). Keeping with the dance example, the PE teacher can ask their children to create dance movement sequences, but this time there is not a predefined music and children’s movement will create music. Each child’s movement is mapped onto a different sound, and children are instructed to create music by combining different movements (for an example of this procedure, see Landry and Jeon, 2017). They are also encouraged to create different combination of sounds by creatively combining movements. By doing this, children have to continuously perceive each movement they perform and regulate the next movement accordingly. This approach will also promote exploration, movement fluency, and functionality, as the produced sound will encourage children to move fluently to “create” a nice and smooth music.

Movement sonification can also motivate performers to pursue new creative movement and increase enjoyment especially in children. Sonification will readily “tell” and reward a performer when a new movement is created and it will encourage children to explore movement in a fun and safe environment. They can play with their movement repertoire *via* the different sounds they can create. Another important aspect worth mentioning is that movement sonification puts performers in charge of the task they are performing. This can likely promote self-regulation (key in embodied cognition, Diamond, 2016; Diamond and Ling, 2020), especially in children, as they have to self-regulate their behavior to keep up with the task and keep the task engaging and fun. Movement sonification will “tell” them straight away if they are disengaging with the task. Lastly, movement sonification can be mapped on movement of each individual, even in a classroom setting, thus it can support the individuality and non-linearity of learning (Newell et al., 2001; Pacheco et al., 2019). The learning intervention will be individualized and will follow the non-linear movement improvement, aligning with the principles of nonlinear pedagogy (Chow et al., 2007, 2015).

Teachers and coaches play a pivotal role in guiding their students toward using sonification for creating original movement. As previously mentioned, they should oversee the creativity process and, if necessary, guide attention to specify knowledge of the environment (Gibson, 1979), this can be done through careful instructions, encouraging their students to explore different and novel movement possibilities. This needs to be done in conjunction with individualizing the movement parameter(s) to sonify. A variety of parameters can be sonified and various sonification techniques have been proposed in the literature (e.g., Hermann et al., 2011; Siegel, 2012). It is beyond the scope of this article to discuss this issue in detail, but we can say that the selection of parameter(s) to sonify is context specific and depends on the teacher’s goal and possibilities (Landry et al., 2014). In a school PE context (as per our example), financial constraints and limited technological expertise may restrict sonification options. However, simple and relatively low-cost strategies can still be implemented. For example, accelerometers placed on pupils’ joints (e.g., wrists and ankles) can sonify movement acceleration, difference in acceleration between body parts, or parts of the body involved in the movement (e.g., see Françoise et al., 2014; Yamaguchi and Kadone, 2017). In such a scenario, the teacher can invite students to explore different movement speed and fluency, and change how they activate the different body parts. Ultimately, schools should not bear the cost of developing a suitable strategy. We presented a principled approach that can underpin the design and development of sonification techniques to influence movement creativity, and we hope that inter-disciplinary collaboration between universities and industry can support schools in the process, as advocated through a transdiscplinary approach by Vaughan et al. (2019).

## Conclusion

In this article, we argue for an embodied approach to creativity that emphasizes the important relationship between movement and cognition in the development of creativity. The development of technologies such as sonification offers new opportunities for designing learning environments that promote creativity. We provided a rationale for using movement sonification to promote creative movement and exemplified its use in creative dance for children. Our approach allows to better understand the embodied nature of creativity as the sonification is “embodied in perception and action” providing a rich landscape for future research to explore creativity. The tasks that can be created can be cognitively challenging and involve a high degree of problem solving that may transfer to more divergent and creative thinking in the classroom.

We contend that learning to explore original dance sequences using movement sonification may provide a meaningful link between creative movement and creative cognition. This association is predicated, in part, on functional similarities between novel actions and thought, such that the process of learning how novel movement parameters map onto sound may facilitate perception-action coupling in novel contexts sharing similar features. In this vein, children may be more inclined to exploit environmental affordances in the classroom after experiencing the self-regulatory process of creating music through physical movement. This may mean that children become more likely to rely on their minds *and* bodies as tools for creative thinking and exploration, such as using their whole bodies to learn the shapes of letters and numbers or acting out scenes from history and science lessons as a strategy for learning new concepts. Future empirical work is necessary to investigate whether and how transfer may unfold from movement sonification to diverse creative problem-solving contexts.

## Data Availability Statement

The original contributions presented in the study are included in the article/supplementary material, further inquiries can be directed to the corresponding author.

## Author Contributions

All authors conceptualized, drafted, edited, reviewed, and approved the manuscript.

### Conflict of Interest

The authors declare that the research was conducted in the absence of any commercial or financial relationships that could be construed as a potential conflict of interest.

## References

[ref1] Amabile T. M. (2018). Creativity in context: Update to the social psychology of creativity. New York: Routledge.

[ref2] Bara F. Bonneton-Bottè N. (2018). Learning letters with the whole body: visuomotor versus visual teaching in kindergarten. Percept. Mot. Skills 125, 190–207. 10.1177/0031512517742284, PMID: 29161949

[ref3] Beghetto R. A. Kaufman J. C. (2010). Nurturing creativity in the classroom. New York: Cambridge University Press.

[ref4] Boocock M. Naudé Y. Taylor S. Kilby J. Mawston G. (2019). Influencing lumbar posture through real-time biofeedback and its effects on the kinematics and kinetics of a repetitive lifting task. Gait Posture 73, 93–100. 10.1016/j.gaitpost.2019.07.127, PMID: 31302338

[ref5] Bruineberg J. Rietveld E. (2014). Self-organization, free energy minimization, and optimal grip on a field of affordances. Front. Hum. Neurosci. 8:599. 10.3389/fnhum.2014.00599, PMID: 25161615 PMC4130179

[ref6] Chemero A. (2003). An outline of a theory of affordances. Ecol. Psychol. 15, 181–195. 10.1207/S15326969ECO1502_5

[ref7] Chow J. Y. Davids K. Button C. Renshaw I. (Eds.) (2015). Nonlinear pedagogy in skill acquisition: An introduction. (London: Routledge).

[ref8] Chow J. Y. Davids K. Renshaw I. Button C. Shuttleworth R. Araújo D. (2007). The role of nonlinear pedagogy in physical education. Rev. Educ. Res. 77, 251–278. 10.3102/003465430305615

[ref9] Craft A. (2003). The limits to creativity in education: dilemmas for the educator. Br. J. Educ. Stud. 51, 113–127. 10.1111/1467-8527.t01-1-00229

[ref10] Cropley A. (2006). Creativity: a social approach. Roeper Rev. 28, 125–130. 10.1080/02783190609554351

[ref11] Dahlstedt P. Dahlstedt A. S. (2019). “OtoKin: mapping for sound space exploration through dance improvisation” in Paper Presented at the International Conference on New Interfaces for Musical Expression; June 3–6, 2019; Brazil.

[ref12] Davids K. Handford C. Williams M. (1994). The natural physical alternative to cognitive theories of motor behaviour: an invitation for interdisciplinary research in sports science? J. Sports Sci. 12, 495–528. 10.1080/02640419408732202 7853448

[ref13] Diamond A. (2016). “Why improving and assessing executive functions early in life is critical” in Executive function in preschool-age children: Integrating measurement, neurodevelopment, and translational research. eds. Griffin J. A. McCardle P. Freund L. S. (Washington: American Psychological Association), 11–43.

[ref14] Diamond A. Ling D. S. (2020). “Review of the evidence on, and fundamental questions about, efforts to improve executive functions, including working memory” in Cognitive and working memory training: Perspectives from psychology, neuroscience, and human development. eds. Novick J. M. Bunting M. F. Dougherty M. R. Randall W. E. (New York: Oxford University Press), 145–389.

[ref15] Diniz N. Coussement P. Deweppe A. Demey M. Leman M. (2012). An embodied music cognition approach to multilevel interactive sonification. J. Multimodal User In. 5, 211–219. 10.1007/s12193-011-0084-2

[ref16] Dyer J. F. Stapleton P. Rodger M. W. M. (2015). Sonification as concurrent augmented feedback for motor skill learning and the importance of mapping design. Open Psychol. J. 8, 192–202. 10.2174/1874350101508010192

[ref17] Dyer J. F. Stapleton P. Rodger M. (2017). Mapping sonification for perception and action in motor skill learning. Front. Neurosci. 11:463. 10.3389/fnins.2017.00463, PMID: 28871218 PMC5566964

[ref18] Effenberg A. O. (2005). Movement sonification: effects on perception and action. IEEE MultiMedia 12, 53–59. 10.1109/MMUL.2005.31

[ref19] Effenberg A. O. Fehse U. Schmitz G. Krueger B. Mechling H. (2016). Movement sonification: effects on motor learning beyond rhythmic adjustments. Front. Neurosci. 10:219. 10.3389/fnins.2016.00219, PMID: 27303255 PMC4883456

[ref20] Erdem C. Schia K. H. Jensenius A. R. (2019). “Vrengt: a shared body-machine instrument for music-dance performance” in Paper Presented at the International Conference on New Interfaces for Musical Expression; June 3–6, 2019; Brazil.

[ref21] Fajen B. R. (2005). Perceiving possibilities for action: on the necessity of calibration and perceptual learning for the visual guidance of action. Perception 34, 717–740. 10.1068/p5405, PMID: 16042193

[ref22] Françoise J. Fdili Alaoui S. Schiphorst T. Bevilacqua F. (2014). “Vocalizing dance movement for interactive sonification of laban effort factors” in Paper Presented at the Proceedings of the 2014 Conference on Designing Interactive Systems; June 21–25, 2014; Vancouver, BC, Canada.

[ref23] Frith E. Loprinzi P. D. Miller S. E. (2019). Role of embodied movement in assessing creative behavior in early childhood: a focused review. Percept. Mot. Skills 126, 1058–1108. 10.1177/0031512519868622, PMID: 31407960

[ref24] Gibson J. J. (1979). The ecological approach to visual perception. Boston: Houghton Mifflin.

[ref25] Glaveanu V. P. Hanchett Hanson M. Baer J. Barbot B. Clapp E. P. Corazza G. E. (2019). Advancing creativity theory and research: a socio-cultural manifesto. J. Creat. Behav. 0, 1–5. 10.1002/jocb.395

[ref26] Hermann T. Hunt A. Neuhoff J. G. (Eds.) (2011). The sonification handbook. (Berlin, Germany: Logos Publishing House).

[ref27] Hristovski R. Davids K. Araujo D. (2009). “Information for regulating action in sport: metastability and emergence of tactical solutions under ecological constraints” in Perspectives on cognition and action in sport. eds. Araujo D. Ripoll H. Raab M. (Hauppauge, NY: Nova Science Publishers), 43–57.

[ref28] Hristovski R. Davids K. Araujo D. Passos P. (2011). Constraints-induced emergence of functional novelty in complex neurobiological systems: a basis for creativity in sport. Nonlinear Dynamics Psychol. Life Sci. 15, 175–206. PMID: 21382260

[ref29] Hristovski R. Davids K. Passos P. Araujo D. (2012). Sport performance as a domain of creative problem solving for self-organizing performer-environment systems. Open Sports Sci. J. 5, 26–35. 10.2174/1875399X01205010026

[ref30] Hutton E. Sundar S. S. (2010). Can video games enhance creativity? Effects of emotion generated by dance dance revolution. Creat. Res. J. 22, 294–303. 10.1080/10400419.2010.503540

[ref31] Jolicoeur P. (1988). Mental rotation and the identification of disoriented objects. Can. J. Psychol. 42, 461–478. 10.1037/h0084200, PMID: 3268325

[ref32] Kelso J. A. S. (2012). Multistability and metastability: understanding dynamic coordination in the brain. Philos. Trans. R. Soc. Lond. Ser. B Biol. Sci. 367, 906–918. 10.1098/rstb.2011.0351, PMID: 22371613 PMC3282307

[ref33] Kimmel M. Hristova D. Kussmaul K. (2018). Sources of embodied creativity: interactivity and ideation in contact improvisation. Behav. Sci. 8:52. 10.3390/bs8060052, PMID: 29882858 PMC6027199

[ref34] Kirk E. Lewis C. (2017). Gesture facilitates children’s creative thinking. Psychol. Sci. 28, 225–232. 10.1177/0956797616679183, PMID: 28182524

[ref35] Kuo C. Y. Yeh Y. Y. (2016). Sensorimotor-conceptual integration in free walking enhances divergent thinking for young and older adults. Front. Psychol. 7:1580. 10.3389/fpsyg.2016.01580, PMID: 27790178 PMC5061809

[ref36] Landry S. Jeon M. (2017). “Participatory design research methodologies: a case study in dancer sonification” in Paper Presented at the 23rd International Conference on Auditory Display; June 19–23, 2017; Pennsylvania, US.

[ref37] Landry S. Ryan J. D. Jeon M. (2014). “Design issues and considerations for dance-based sonification” in Paper Presented at the 20th International Conference on Auditory Display. June 22–25, 2017. New York, USA.

[ref38] Lem A. Paine G. Drummond J. (2010). “A dynamic sonification device in improvisational music therapy” in Paper Presented at the 4th International Technology, Education and Development Conference; March 8–10, 2010; Spain.

[ref39] Leung A. K. Y. Kim S. Polman E. Ong L. Qiu L. (2012). Embodied metaphors and creative “acts”. Psychol. Sci. 23, 502–509. 10.1177/0956797611429801, PMID: 22477105

[ref40] Lorenzoni V. Staley J. Marchant T. Onderdijk K. E. Maes P. J. Leman M. (2019). The sonic instructor: a music-based biofeedback system for improving weightlifting technique. PLoS One 14:e0220915. 10.1371/journal.pone.0220915, PMID: 31461448 PMC6713320

[ref41] Lubart T. I. Georgsdottir A. (2004). “Creativity: developmental and cross-cultural issues” in Creativity: When east meets west. eds. Lau S. Hui A. N. N. Ng G. Y. C. (River Edge, NJ: World Scientific Publishing Company), 23–54.

[ref42] Memmert D. Perl J. (2009). Analysis and simulation of creativity learning by means of artificial neural networks. Hum. Mov. Sci. 28, 263–282. 10.1016/j.humov.2008.07.006, PMID: 19110331

[ref43] Michaels C. Beek P. (1995). The state of ecological psychology. Ecol. Psychol. 7, 259–278. 10.1207/s15326969eco0704_2

[ref44] Moran S. (2010). “The roles of creativity in society” in The Cambridge handbook of creativity. eds. Kaufman J. C. Sternberg R. J. (New York, NY: Cambridge University Press), 74–90.

[ref45] Newell K. M. Liu Y. Mayer-Kress G. (2001). Time scales in motor learning and development. Psychol. Rev. 108, 57–82. 10.1037/0033-295X.108.1.57, PMID: 11212633

[ref46] Orth D. van der Kamp J. Memmert D. Savelsbergh G. J. P. (2017). Creative motor actions as emerging from movement variability. Front. Psychol. 8:1903. 10.3389/fpsyg.2017.01903, PMID: 29163284 PMC5671646

[ref47] Pacheco M. M. Lafe C. W. Newell K. M. (2019). Search strategies in the perceptual-motor workspace and the acquisition of coordination, control, and skill. Front. Psychol. 10:1874. 10.3389/fpsyg.2019.01874, PMID: 31474912 PMC6702327

[ref48] Pllana D. (2019). Creativity in modern education. World J. Educ. 9, 136–140. 10.5430/wje.v9n2p136

[ref49] Rasmussen L. J. T. Østergaard L. D. Glăveanu V. P. (2017). Creativity as a developmental resource in sport training activities. Sport Educ. Soc. 24, 491–506. 10.1080/13573322.2017.1403895

[ref50] Richards R. (2010). “Everyday creativity” in The Cambridge handbook of creativity. eds. Kaufman J. C. Sternberg R. J. (New York, NY: Cambridge University Press), 189–215.

[ref51] Rietveld E. Kiverstein J. (2014). A rich landscape of affordances. Ecol. Psychol. 26, 325–352. 10.1080/10407413.2014.958035

[ref52] Risko E. F. Gilbert S. J. (2016). Cognitive offloading. Trends Cogn. Sci. 20, 676–688. 10.1016/j.tics.2016.07.002, PMID: 27542527

[ref53] Rizzo A. El Raheb K. Cisneros R. E. K. Whatley S. Zanoni M. Camurri A. (2018). “WhoLoDancE: whole-body interaction learning for dance education” in Paper Presented at the EUROMED International Conference on Digital Heritage; October 29 – November 3, 2018; Cyprus.

[ref54] Rudd J. R. Pesce C. Strafford B. W. Davids K. (2020). Physical literacy, a journey of individual enrichment: an ecological dynamics rationale for enhancing performance and physical activity in all. Front. Psychol. 11:1904. 10.3389/fpsyg.2020.01904, PMID: 32849114 PMC7399225

[ref55] Runco M. A. (2014). “Big C, little c” creativity as a false dichotomy: reality is not categorical. Creat. Res. J. 26, 131–132. 10.1080/10400419.2014.873676

[ref56] Runco M. A. Jaeger G. J. (2012). The standard definition of creativity. Creat. Res. J. 24, 92–96. 10.1080/10400419.2012.650092

[ref57] Schaffert N. Janzen T. B. Mattes K. Thaut M. H. (2019). A review on the relationship between sound and movement in sports and rehabilitation. Front. Psychol. 10:244. 10.3389/fpsyg.2019.00244, PMID: 30809175 PMC6379478

[ref58] Seifert L. Button C. Davids K. (2013). Key properties of expert movement systems in sport: an ecological dynamics perspective. Sports Med. 43, 167–178. 10.1007/s40279-012-0011-z, PMID: 23329604

[ref59] Shapiro L. Stolz S. A. (2019). Embodied cognition and its significance for education. Theory Res. Educ. 17, 19–39. 10.1177/1477878518822149

[ref60] Siegel W. (2012). “Dancing the music: interactive dance and music” in The Oxford handbook of computer music. ed. Dean R. T. (Oxford, UK: Oxford University Press).

[ref61] Sigrist R. Rauter G. Riener R. Wolf P. (2013). Augmented visual, auditory, haptic, and multimodal feedback in motor learning: a review. Psychon. Bull. Rev. 20, 21–53. 10.3758/s13423-012-0333-8, PMID: 23132605

[ref62] Slepian M. L. Ambady N. (2012). Fluid movement and creativity. J. Exp. Psychol. Gen. 141, 625–629. 10.1037/a0027395, PMID: 22352395

[ref63] Stanton T. R. Spence C. (2020). The influence of auditory cues on bodily and movement perception. Front. Psychol. 10:3001. 10.3389/fpsyg.2019.03001, PMID: 32010030 PMC6978806

[ref64] Sternberg R. J. Lubart T. I. (1999). “The concept of creativity: prospects and paradigms” in Handbook of creativity. ed. Sternberg R. J. (New York, NY: Cambridge University Press), 3–15.

[ref65] Torrents C. Balagué N. Ric Á. Hristovski R. (2020). The motor creativity paradox: constraining to release degrees of freedom. Psychol. Aesthet. Creat. Arts. 10.1037/aca0000291 [Epub ahead of print]

[ref66] Torrents C. Ric Á. Hristovski R. (2015). Creativity and emergence of specific dance movements using instructional constraints. Psychol. Aesthet. Creat. Arts 9, 65–74. 10.1037/a0038706

[ref67] Torrents C. Ric A. Hristovski R. Torres-Ronda L. Vicente E. Sampaio J. (2016). Emergence of exploratory, technical and tactical behavior in small-sided soccer games when manipulating the number of teammates and opponents. PLoS One 11:e0168866. 10.1371/journal.pone.0168866, PMID: 28005978 PMC5179131

[ref68] Tversky B. (2009). “Spatial cognition: embodied and situated” in Cambridge handbook of situated cognition. eds. Robbins P. Aydede M. (Cambridge: Cambridge University Press), 201–216.

[ref69] van Dijk L. Rietveld E. (2016). Foregrounding sociomaterial practice in our understanding of affordances: the skilled intentionality framework. Front. Psychol. 7:1969. 10.3389/fpsyg.2016.01969, PMID: 28119638 PMC5220071

[ref70] Vaughan J. Mallett C. J. Davids K. Potrac P. Lopez-Felip M. A. (2019). Developing creativity to enhance human potential in sport: a wicked transdisciplinary challenge. Front. Psychol. 10:2090. 10.3389/fpsyg.2019.02090, PMID: 31572271 PMC6753247

[ref71] Ward T. B. Smith S. M. Finke R. A. (1999). “Creative cognition” in Handbook of creativity. ed. Sternberg R. J. (New York, NY: Cambridge University Press), 189–212.

[ref72] Warren W. H. (2006). The dynamics of perception and action. Psychol. Rev. 113, 358–389. 10.1037/0033-295X.113.2.358, PMID: 16637765

[ref73] Wood K. Cisneros R. E. Whatley S. (2017). Motion capturing emotions. Open Cult. Stud. 1, 504–513. 10.1515/culture-2017-0047

[ref74] Woods C. T. McKeown I. Rothwell M. Araujo D. Robertson S. Davids K. (2020). Sport practitioners as sport ecology designers: how ecological dynamics has progressively changed perceptions of skill ‘acquisition’ in the sporting habitat. Front. Psychol. 11:654. 10.3389/fpsyg.2020.00654, PMID: 32390904 PMC7194200

[ref75] Wulf G. (2013). Attentional focus and motor learning: a review of 15 years. Int. Rev. Sport Exerc. Psychol. 6, 77–104. 10.1080/1750984X.2012.723728

[ref76] Wyrick W. (1968). The development of a test of motor creativity. Restor. Q. 39, 756–765. 10.1080/10671188.1968.10616608, PMID: 5246984

[ref77] Yamaguchi T. Kadone H. (2017). Bodily expression support for creative dance education by grasping-type musical interface with embedded motion and grasp sensors. Sensors 17:1171. 10.3390/s17051171, PMID: 28531114 PMC5470916

[ref78] Zhou Y. Zhang Y. Hommel B. Zhang H. (2017). The impact of bodily states on divergent thinking: evidence for a control-depletion account. Front. Psychol. 8:1546. 10.3389/fpsyg.2017.01546, PMID: 29033862 PMC5626876

